# The Hip Instructional Prehabilitation Program for Enhanced Recovery (HIPPER) as an eHealth Approach to Presurgical Hip Replacement Education: Protocol for a Randomized Controlled Trial

**DOI:** 10.2196/29322

**Published:** 2021-07-06

**Authors:** William C Miller, Somayyeh Mohammadi, Wendy Watson, Morag Crocker, Marie Westby

**Affiliations:** 1 GF Strong Rehabilitation Research Program Vancouver, BC Canada; 2 Department of Occupational Science and Occupational Therapy Faculty of Medicine University of British Columbia Vancouver, BC Canada; 3 Vancouver Coastal Health Vancouver, BC Canada

**Keywords:** total hip replacement, osteoarthritis, eHealth, prehabiliatation, preoperative education, randomized controlled trial, evaluation, feasibility, rehabilitation, recovery, hip, bone, surgery, education

## Abstract

**Background:**

Osteoarthritis (OA), leading to hip replacement (THR), is a primary contributor to global mobility impairment. In 2018, more than 59,000 THR surgeries were performed in Canada. Health promotion education, such as prehabilitation, is vital to optimizing surgical outcomes.

**Objective:**

This study aims to evaluate the feasibility of the Hip Instructional Prehabilitation Program for Enhanced Recovery (HIPPER), an eHealth approach to prehabilitation education.

**Methods:**

A single-blind (assessor-blind), 2-arm, feasibility randomized controlled trial will be conducted. We will recruit 40 (HIPPER group, n=20; control group, n=20) older adults with hip OA and on a waitlist for a THR. The HIPPER intervention consists of 12 online, interactive modules. The control group will receive the current standard practice consisting of 2 online educational sessions lasting 2 hours each (webinars). Feasibility outcomes (eg, recruitment and retention rates) will be evaluated.

**Results:**

Recruitment started in March 2021. As of April 20, 2021, 18 participants were recruited. All 18 completed T1 measures. Only 1 participant has been scheduled to have a surgery and therefore has been scheduled to complete T2 measures. The remainder of the participants are waiting to be notified of their surgery date. This project was funded by a Canadian Institutes of Health Research Project Grant. Our institute’s research ethics board approved this study in November 2016.

**Conclusions:**

Results will lead to refinement of the HIPPER protocol in order to evaluate a standardized and geographically accessible prehabilitation program.

**Trial Registration:**

ClinicalTrials.gov NCT02969512; https://clinicaltrials.gov/ct2/show/NCT02969512

**International Registered Report Identifier (IRRID):**

DERR1-10.2196/29322

## Introduction

Total hip replacements (THRs) reduce joint pain and improve function in individuals with advanced osteoarthritis (OA). The incidence of THRs has grown over the last 5 years (~17.4%), with 59,000 THRs in Canada between 2017 and 2018, resulting in substantial health care costs [1]. Health promotion education provided prior to surgery can reduce direct and indirect costs [2], improve patient care and recovery [3-5], reduce hospitalization costs [6], accelerate rehabilitation [7,8], and reduce preoperative pain and anxiety [3,4]. It is thus a valid indicator of quality rehabilitation [9].

Provision of preoperative education and access to rehabilitation prior to THR are inconsistent and sometimes nonexistent in parts of British Columbia (BC). Given the majority of THRs are performed in southern urban centers, individuals from remote areas often travel great distances to receive prehabilitation (“prehab”) — presurgery education and exercise training — or receive it immediately prior to surgery, reducing many benefits (eg, prehab exercise to maintain function). Prehab is often limited to written materials or didactic group sessions [8,10]. While the literature suggests prehab may be beneficial, study results are inconsistent, and effect sizes for the most prominent outcome (anxiety) are modest at best [8,10]. We hypothesize that this is due to issues with educational content or delivery and study design error with measuring the correct outcomes at the right time. For example, the assertion that prehab reduces anxiety and maintains function should be measured immediately before surgery.

Health education using eHealth (eg, delivered online) approaches have been lauded for being interactive and enabling learners to re-engage over sustained periods [11-13]. eHealth has the potential to improve quality of care for older adults [14], enhance communication between patients and health care providers, reduce costs, and increase access to health care and information [15]. For eHealth to be effective, understanding patient technology preferences, including adoption facilitators and barriers, is required [14]. Though less likely to embrace eHealth compared to their younger counterparts, computer use among older adults in BC continues to grow, with 86% of those 45-64 years old and 55% of those >65 years old using the internet from home [16].

For these reasons, we developed HIPPER (Hip Instructional Prehabilitation Program for Enhanced Recovery), an interactive, user-centered, eHealth, preoperative, THR education program based on adult learning principles [17,18]. HIPPER is a potential model for use across diagnostic groups (eg, knee or shoulder replacement), providing standardized, user-friendly prehab, reducing the time clinicians must spend on providing prehab education, decreasing the time patients spend on traveling to access in-person education, and leading to reduction in direct health care costs and indirect costs (eg, dependence on family). Finally, HIPPER has the potential to allow participants to personalize prehab education based on their needs, access prehab education at any time, review prehab education multiple times, and test their knowledge.

We will conduct a 2-year, single-blinded, feasibility, randomized controlled trial (RCT) to address the design and HIPPER intervention fidelity. The primary feasibility objectives are to assess (1) process issues (eg, participant recruitment, retention, perceived benefit), (2) resource issues (eg, treatment adherence and burden), (3) management issues (eg, participant processing, protocol administration), and (4) treatment issues (eg, safety, treatment effect). See Table 1. Secondary objectives are to evaluate the effect of HIPPER on primary and secondary clinical outcomes and obtain an estimate of treatment effect size. Specifically, we hypothesized that, in patients undergoing hip replacement surgery, using HIPPER is as effective as usual care in reducing preoperative anxiety. In addition, we hypothesized that, in patients undergoing hip replacement surgery, using HIPPER is as effective as usual care in improving physical function, self-efficacy, and health-related quality of life 1 month and 3 months after surgery.

**Table 1:** Feasibility indicators.

Feasibility component		Indicator	Criteria
**Process**			
	Recruitment rate	Number of participants recruited; number of women and men recruited	Mean of 4 participants/month: total of 44 over 11 months
	Consent rate	% of participants consenting	<10% participant refusal
	Retention rate	% of participants with T4^a^ data	Complete data collection for >80%
	Perceived benefit	Posttreatment participant questionnaire; qualitative interviews at T4	>85% of responses will be “strongly agree/agree”; qualitative analysis will inform clinical importance
	Assessor masking	% unaware of group status	100% of participants do not unmask their treatment
**Resources**			
	Treatment adherence	HIPPER^b^ group spends 2.5 hours on all modules; control group attends both prehab education sessions	>85% of participants
	Data collection (T): participant & assessor burden	T1 duration; T2, T3, & T4 durations	>85% of participants complete in ≤2 hours; >85% of participants complete in ≤1.5 hours
	Collection of EQ-5D^c^ data	Administration; EQ-5D pre/post score	Mean EQ-5D administration is <10 minutes; statistically significant change between T1 & T2
	Educator burden	Time (minutes) spent in answering participants’ questions and following up with them	Mean time spent per participant is <2 hours for T1 and <1 hour for T2; <20% phone call back for clarification
**Management**			
	Internet stability	Downtime due to technical or mechanical issues	>90% of participants are not without internet for >2 days
	Participant processing time	Time from data collection to treatment	Mean time is <10 days at each site
	Treatment administration issues	Post-treatment evaluation form (study educator)	Any issues identified modifiable without substantial changes to the protocol
**Treatment**			
	Safety (data collection & training)	Adverse events during assessment or training	No major injuries nor adverse events (eg, dislocation) reported
	Dose level response	Correlation between training time and change score	Minimum practice time guidelines sufficient for a treatment effect

^a^Fourth measurement timepoint.

^b^HIPPER: Hip Instructional Prehabilitation Program for Enhanced Recovery.

^c^EQ-5D: EuroQol 5 Dimension.

## Methods

This protocol has been written based on SPIRIT (Standard Protocol Items: Recommendations for International Trials) [19].

### Trial Design

An equivalence, single-blind (assessor-blind), 2-arm, feasibility RCT with a 1:1 allocation ratio will be conducted to compare an eHealth program to standard care for people undergoing elective THR.

### Study Setting

Patients referred to the OsteoArthritis Service Integration System (OASIS) program (n>250/year), a central intake program administered by Vancouver Coastal Health in Canada, will be approached to participate.

### Eligibility Criteria

#### Inclusion Criteria

To be included, participants will be ≥50 years old, have hip OA, be scheduled to have a single THR in >12 weeks, and have internet access.

#### Exclusion Criteria

Individuals who cannot communicate and complete questionnaires in English, anticipate a health condition or procedure that may result in cancelation of their THR surgery, are actively receiving physical therapy for their hip symptoms, have had a previous THR (on either side), or have already received prehab education will be excluded.

### Interventions

#### Standard Care

To provide a comparable level of education, control participants will receive 2-hour educational live webinars, as per current practice in OASIS. The webinar focuses on prehab material (eg, exercise, nutrition) and presurgical content (eg, home equipment, hip precautions). The participants will be able to have their questions addressed throughout the live webinars. In addition, the participants will receive an educational booklet that provides information on various topics related to their hip surgery such as exercise, preparing their home, and an equipment checklist. Participants in this group are verbally encouraged to take part in the live webinars.

#### Intervention

As a team, we created HIPPER with a user-centered approach consisting of focus groups and a “think aloud” cognitive processing technique, both of which provided improved usability. HIPPER consists of 12 interactive online modules (~20 minutes each), which permit self-paced progression and access from the participant’s home or location of choice. HIPPER participants will be contacted by email or phone to provide them with website portal access and simple instructions using personalized encrypted login information. Upon login, the participants can immediately begin the modules. Participants can stop a module at any point, and their progress will be saved. When ready, they can continue where they left off or choose to go backward and review previous content. Modules are not “marked” complete until the participant hits the finished icon at its end. The module format includes embedded videos, narrated slides, and quizzes. Participants will be encouraged to have a family member view HIPPER with them. An education consultant (with expertise in designing courses with Articulate Storyline 360) will monitor and troubleshoot any platform issues throughout the study. The education consists of a comprehensive library of material addressing topics such as exercise, equipment needs and setup, pain management, nutrition, and weight management. The research coordinator who can remotely monitor online analytics (eg, login frequency, module progression) will phone the participant within 2 weeks if no online activity is noted to promote adherence and troubleshoot potential technical problems. The participant will be given a phone number and email to contact the research coordinator should they experience difficulty using the modules.

Participants in the HIPPER group will be asked not to attend the OASIS live webinars on prehab education. The research coordinator will send the name of the participants in the HIPPER group to staff at OASIS who can monitor whether HIPPER participants attended the online webinars.

Participants in both groups can withdraw from the study at any time. Some participants in the HIPPER group may gain access to the educational booklet through the surgery office or other sources. Having access to the educational booklet will not make the participants ineligible from participating in the HIPPER group.

### Outcome Measures

#### Primary Outcome

The primary outcome will be measured using the Hospital Anxiety and Depression Scale (HADS-A)**.** Duivenvoorden and colleagues [20] have shown that at least 30% of patients undergoing THR surgery experience clinically significant levels of anxiety (treated by a psychiatrist or a psychologist for their anxiety). However, studies in other patient populations suggest as much as 50% to 80% of patients experience moderate to high levels of anxiety prior to major elective surgeries. Systematic reviews [8,10] report consistent and clinically meaningful improvements in preoperative anxiety with prehab education [9]. Therefore, preoperative anxiety was selected as our primary outcome in this feasibility study.

#### Secondary Outcomes

Given the internationally generated and recommended core self-report [21] and performance-based [22] measures for OA, recently published rehabilitation quality indicators [9] and the existing literature demonstrating the relationship between pain, physical function or activity, and self-efficacy on THR outcomes, a number of secondary measures will be administered.

The first secondary outcome will be measured using the Oxford Hip Score (OHS). The OHS is a 12-item tool that assesses pain and function in patients undergoing THR surgery. It demonstrates good construct validity and test-retest reliability in THR [23,24]. Participants will complete this measure at all time points (T1-T4).

The second secondary outcome will be measured using the 30-second Chair Stand Test (30-sec CST). Functional lower limb strength and dynamic balance will be assessed by having participants perform repeated sit-to-stands using a standard 43-46 cm straight-back chair with no arm rests. Excellent test-retest reliability has been reported in patients awaiting THR [25]. This test has been proven to have acceptable validity and high correlations with other measures of physical functions in patients with joint replacement [26]. Participants will be asked to complete this test at T1, T2, and T4.

The third secondary outcome will be measured with the Physical Activity Scale for the Elderly (PASE). The PASE is a 12-item tool developed for older adults to assess home, occupational, and recreational activities in the previous 7 days [27]. It has moderate test-retest reliability (intraclass correlation coefficient=.77) in hip OA and correlates well with other self-report activity measures [28]. The intraclass correlation coefficient for the entire scale has been found to be .78 [28]. Participants will complete this measure at T1-T4.

The fourth secondary outcome will be measured using the Self-Efficacy for Rehabilitation Outcome Scale (SER). The SER is a 12-item questionnaire developed for patients undergoing hip or knee surgery and asks patients to rate their confidence on an 11-point Likert scale. It generates 2 subscale scores: self-efficacy for rehabilitation therapy exercises and self-efficacy for overcoming barriers [29]. The Cronbach alpha for the SER was .94 [30]. Participants will complete this measure at T1-T4.

The fifth secondary outcome will be measured using an equipment checklist. Patients acquire equipment and mobility aids prior to THR to ensure their safety and enable them to carry out activities of daily living [9]. A comprehensive checklist of recommended equipment will be created based on current guidelines, clinical recommendations, and our patient partners’ input. Patients will use the checklist at T2-T4 to record the number and type of equipment items they have used and how often they have used each (eg, dressing equipment). Participants will complete this measure at T2-T4.

The sixth secondary outcome will be measured using the EuroQoL-5 Dimension, 5 Level (EQ-5D-5L). Health-related quality of life is a core outcome for hip OA [21], and its measurement is a recommended quality indicator [9]. The EQ-5D-5L is a brief and well-validated questionnaire that assesses 5 health status domains (mobility, self-care, usual activities, pain/discomfort, and anxiety/depression) [21,31]. Participants will complete this measure at T1-T4.

The final secondary outcome will be measured using the System Usability Scale. This scale consists of a 10-item questionnaire with 5 response options, ranging from “Strongly agree” to “Strongly disagree.” Originally created by Brooke [32], it enables evaluation of a wide variety of products and services, including hardware, software, mobile devices, websites, and applications. Some examples are, “I think that I would like to use this system frequently.” and “I needed to learn a lot of things before I could get going with this system.” In this study, we replaced “the system” with “HIPPER” to capture the opinion of participants regarding the usability of our eHealth program. Participants will complete this measure at T2-T4 (if randomized to the treatment group).

### Participant Timeline

**Figure 1:**
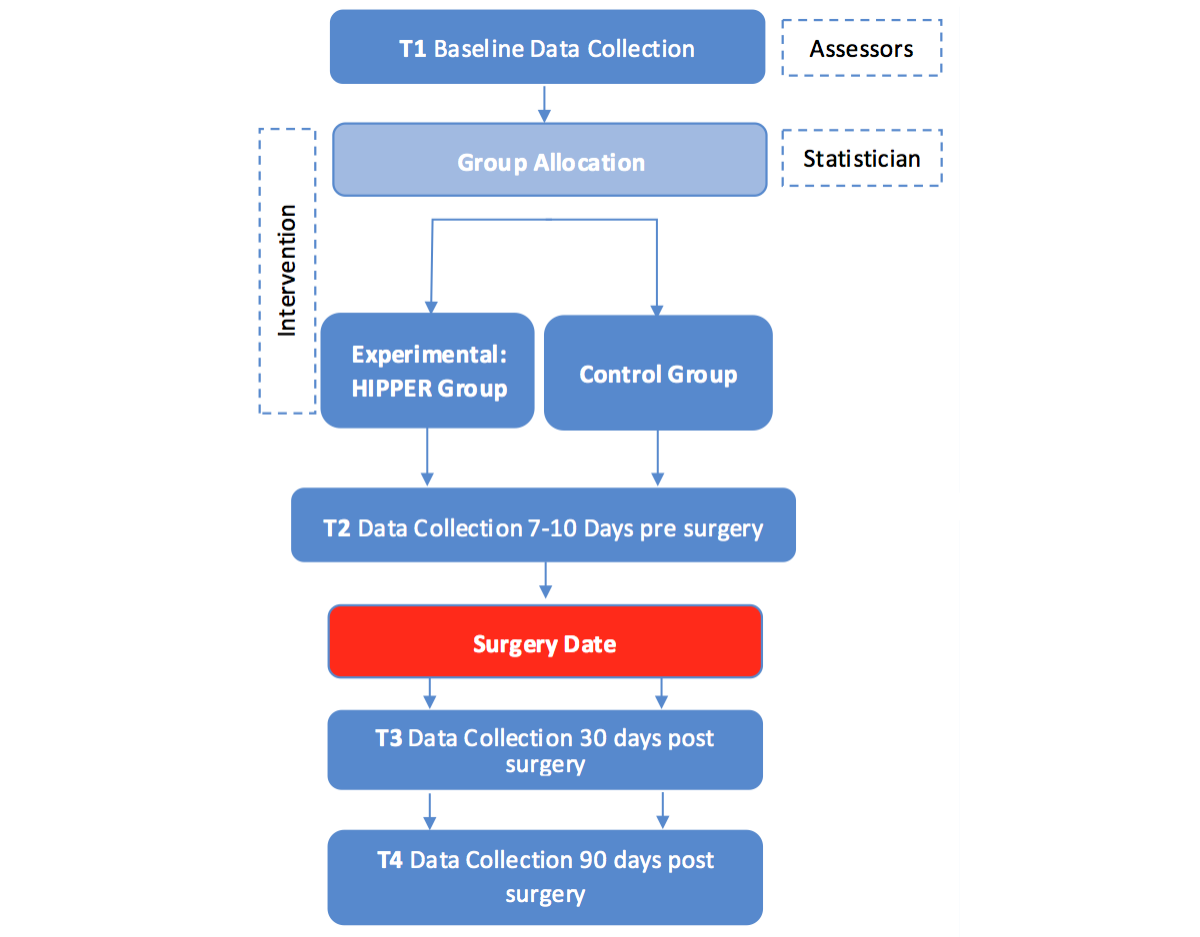
Data collection procedure for the randomized controlled trial. HIPPER: Hip Instructional Prehabilitation Program for Enhanced Recovery.

T1 data will be collected at approximately 12 weeks before the THR surgery. Follow-up data will be collected 7-10 days prior to surgery (T2), 30 days (T3) postsurgery, and 90 days (T4) postsurgery. Figure 1 shows the participant timeline.

### Sample Size

The suggested sample size for feasibility RCTs ranges from 12 to 30 per arm [33,34]; therefore, we aim to recruit 40 participants and randomly assign them to the HIPPER group or control group (usual care). Following other feasibility RCTs (eg, [35,36]), descriptive information on feasibility outcomes and means, SDs, and Cohen d effect sizes will be provided to be used in the future multisite RCT.

The feasibility RCT is a vital step to estimate the parameters needed for designing a full-scale RCT [37] that selects the right outcome measures, fidelity of the protocol and randomization, feasibility of recruitment, adherence and response rate, and feasibility of quantitative measures.

### Recruitment

The OASIS will send mail to or email patients who are on the waitlist for THR. Patients will be encouraged to contact the researchers to learn more about the study.

### Data Collection Method and Data Management

Our collaborators at OASIS will send mail to or email the patients who are on a waiting list to attend prehab classes. Interested patients will be encouraged to contact our research center. They will be interviewed by the assessor to determine eligibility. Upon successful screening, participants will be sent a PDF version of the consent form to keep for their record and also a link to the online consent form on Qualtrics, a secure data collection tool. Participants will be asked to sign the online consent form. Participants will be given at least 24 hours to read the consent form before attending the T1 data collection online meeting. After signing the consent form, participants will be asked to attend the T1 data collection online meeting. During this initial meeting, the assessor will help them complete a set of questionnaires using Qualtrics. In addition to the self-reported measures, the assessor will ask participants to complete the 30-sec CST, which is a performance test. Participants will be asked to complete the test at home while communicating with the research coordinator as part of the online meeting. Participants will be video recorded during the 30-sec CST. Immediately after they complete the T1 measures and the 30-sec CST, the assessor will leave the meeting, and the research coordinator will join the participant and use secure online software [38] to randomize the participant. The assessor will remain blind to the randomization result.

To collect the data at T2, T3, and T4, the assessor will send a link to the participants to complete the questionnaires in Qualtrics. In addition, at T2 and T4, the assessor will contact the participants to schedule a short online meeting during which the participants will be asked to do the 30-sec CST via videoconference. The assessor will ask the participants to not reveal their group allocation to them.

### Allocation

After participants complete the T1 measures, the assessor will invite the research coordinator to join the Zoom meeting; then, the assessor will leave the meeting. During the meeting, the research coordinator will use the Simple Randomization Service provided by Sealed Envelope [38] to randomize the participant into the HIPPER or control group. The research coordinator will add the participant ID to the account that we created for this trial and then click on the randomize button. Then, the Simple Randomization Service provided by Sealed Envelope [38] will randomize participants into either group A (HIPPER) or B (Control). We will not stratify the sample. Block randomization will be used to ensure equal number of participants will be in each group. The assessor will remain blind to participants’ group allocations. After randomization, the research coordinator will give participants instructions based on their group. In addition, the research coordinator will ask the participants to not reveal their group to the assessor.

### Masking

Given the nature of the study, it is not possible to blind the participants and the research coordinator to the participant’s group after baseline data collection. However, the assessor will remain blinded throughout the study. The assessor will be responsible for setting up the Qualtrics measures for the participants. In Qualtrics, the survey has been set up with only one arm; therefore, the assessor will not need the participant’s group to schedule the invitations.

After baseline data collection, the assessor will only contact participants to remind them to fill out the questionnaires and perform the 30-sec CST. To address performance bias, participants will be instructed not to discuss their group allocation, and the assessor will reinforce this point before starting each data collection session. The assessors will be asked to track and report any unblinding. If unblinding occurs before the data collection for that session, the assessor will reschedule the session, and another assessor will complete the data collection. If unblinding happens after the data collection at T2 or T3, another assessor will be assigned for the remaining data collection session(s). Using patient-reported and standardized objective measures also decreases risk of performance bias. In addition, the assessor will video record the the 30-sec CST. This test will be scored by the assessor and another researcher (who has not been involved in performing the 30-sec CST). If there is a discrepancy between the assessor’s and the other researcher’s score on the 30-sec CST, a third researcher will be involved to score the 30-sec CST. This will prevent any bias in scoring the 30-sec CST that may arise from the assessor’s assumptions about the participant’s group allocation.

### Statistical Methods

#### Analyses

Analyses will consider study feasibility as well as clinical (statistical) outcomes. Distribution of data will be evaluated by applying the one-sample Kolmogorov-Smirnov test. If data are found to be normally distributed, means and SDs (continuous variables) and frequencies and proportions (categorical variables) will be used to summarize demographic and outcome variables by groups. Descriptive statistics will be used to describe the sample and to assess online usage data (eg, time spent using modules) to evaluate dose response and adherence for the HIPPER group.

#### Missing Data

Similar to previous research on eHealth education (eg, [39]), missing data will be imputed using multiple imputation, and therefore, intention-to-treat analyses will be used. However, additional participants will be recruited to keep the sample size with complete data at 20 per arm.

#### Feasibility Indicators

The specific feasibility objectives (see Table 1) will be considered “successful,” indicating a sufficiently robust protocol (small or no modification required), or “revise” if a substantive change is required prior to proceeding to a definitive RCT.

#### Clinical Outcomes

Primary and secondary outcomes at T2 will be compared between the HIPPER and control groups using analysis of covariance (ANCOVA), controlling for baseline score as a covariate [40]. Unequal cell sizes will be accommodated using Method 1 adjustment [41,42], and diagnostic assessments will be made for model assumptions. Statistical significance testing (*P*) and marginal means with 95% confidence intervals will be estimated with the alternative hypothesis favoring the HIPPER group. Effect size (partial 2) will be calculated as a ratio of the effect and total sums of squares, with a 95% confidence interval. To preserve prognostic balance, primary analysis will be intention-to-treat, but we will also consider per-protocol analyses as a secondary approach given one objective is to estimate the treatment effect [43]. The focus on a single primary variable indicates we will not control for multiple comparison, as the secondary outcomes are considered exploratory.

### Data Monitoring

A Data and Safety Monitoring Board (statistician, occupational therapist/physical therapist, person with a THR) will review outcome data and advise the team on safety and any need to modify the study design [44]. The Data and Safety Monitoring Board is independent from the funding agency. More information about the board can be found by contacting the primary investigator.

Interim analyses will be conducted after the first 10 participants complete T2 and after the first 10 participants complete T3.

HIPPER incorporates extensive safety-related material including modified home setup (eg, elimination of scatter rugs) in the educational modules. However, adverse events (eg, falls during the 30-CST test) will be recorded and will be reported to the investigators. This process will be independent from the funding agency. In the very unlikely circumstance in which participants experience a decrease in function, substantial injury, or discomfort from their activities in this study, the investigators or study trainers will contact all participants by phone or email and inform them the study is being stopped and explain the reason. 

### Ethics and Dissemination

Ethics approval was obtained from our university Research Ethics Board (H16-02553) and the Vancouver Coastal Health Research Institute (V16-02553). Any changes in the ethics will be submitted as an amendment to our Research Ethics Board. The assessor will be responsible for collecting consent forms from the participants. A PDF version of the consent form and a link to the online version of the consent form will be sent to the participants at least 24 hours before the T1 appointment. Participant will be given the option to either e-sign the PDF version or add their signature to the online consent form.

Email addresses will be captured because the measures should be sent to the same email address throughout the study; however, when conducting the analyses, all identifiable information will be removed from the data. The e-signed consent forms will be received through an email address provided by our research institute that will only be used for this project. Videos of participants will be labeled using a combination of their participant number and time point. Videos will be zipped, and we will add a password to the zipped file to ensure their security. The consent forms and videos will be password protected and will be saved in our lab drive, which is located on our research institute’s servers in a separate folder than the data and the participants’ contact list. The main participants’ data will be collected with the University of British Columbia Qualtrics, and therefore, the servers are located in Canada. 

The corresponding author will be responsible for the data for at least 5 years after the work is published or otherwise presented. At the end of this 5-year period, the paper copies will be shredded using a bonded company, and the computer files will be deleted. The videos will be zipped, and the zipped filed will be password protected. All videos will be saved in a separate folder on an encrypted and password-protected computer. Specifically, the videos will be only saved on our lab’s drive, which is managed by our research institute’s information technology department. All members of the study team will have access to the data. The list of the study team members can be found by contacting the corresponding author.

Our knowledge translation plan will target clinicians both nationally and internationally (clinicians’ feedback will be used to further improve HIPPER), leveraging existing communication tools such as websites (eg, health authorities in BC), electronic and print newsletters (eg, Physiotherapy Association of BC), presentations at provincial practice forums, webinars for rural clinicians, and social media (eg, Twitter). Building on existing partnerships with Bone and Joint Canada and the Arthritis Health Professions Association, a summary will be prepared for their websites and electronic newsletters to reach a national audience. Abstracts will be submitted to conferences (eg, Canadian Orthopaedic Association Meeting). Manuscripts will be submitted for publication. If HIPPER is effective, we will collaborate with OASIS and will allow HIPPER to be hosted on the OASIS website [45]. OASIS and VCH will be responsible for updating and maintaining the content of HIPPER after the end of the research study.

There is no plan to use professional writers for disseminating the findings of this study. Participants who are interested in finding out about the results of this study will be sent a lay summary of the findings. The protocol of this study will be available to anyone interested.

## Results

Recruitment started in March 2021. As of April 20, 2021, 18 participants were recruited. All 18 completed T1 measures. Only 1 participant has been scheduled to have surgery and therefore has been scheduled to complete T2 measures. The remainder of the participants are waiting to be notified of their surgery date. This project was funded by a Canadian Institutes of Health Research Project Grant. Our institute’s research ethics board approved this study in November 2016.

## Discussion

### Potential Impact and Significance of the Study

eHealth is a promising way to address 2 substantial weaknesses in most prehab and presurgical programs, as it can (1) offer learning that is interactive (unlike printed materials) and (2) enable learners to engage over sustained periods (unlike single group sessions). Moreover, eHealth has the potential to improve quality of care for older adults [12,13,46], enhance communication between patients and health care providers [14], reduce costs, and increase access to evidence-based health information. Recent studies have shown that online interventions can substantially increase older adults’ well-being [47] and physical activity levels [48]. In addition, available data show that internet use is growing rapidly among older Canadians. In BC alone, 86% of those aged 45-64 years make up the fastest growing cohort of individuals who need THR [49]. Considering the potential benefits of online education for older adults and the increase in the use of technology, it is reasonable and essential to develop eHealth tools for this population.

### Clinical Contribution

HIPPER was developed with the aim to *improve access* to existing knowledge for patients and their families and *provide customized* (eg, through understanding patients’ perspectives and having a digital interaction component), *interactive* (eg, quizzes), and *engaging eHealth education* (eg, using educational videos and audio) to better prepare patients for THR. In addition, HIPPER will *increase patient knowledge* and health literacy by facilitating access to prehab education. HIPPER will enable *self-paced learning*, with the support of family caregivers and from the comfort of the patient’s home, thus *reducing the burden* on patients, families, and health care providers. It will also allow for *standardized information* to be delivered regardless of geographical boundaries. In contrast to written educational materials, HIPPER’s content can be quickly updated with *minimal financial burden* on the health care system. Finally, it will reduce direct clinician contact and travel demands, leading to a reduction in both direct and indirect health care costs.

### Strengths and Weaknesses of the Study

The results of this feasibility study cannot be generalized to all patients who are preparing for THR. However, considering that more than 85% of patients who undergo hip replacement are >50 years old [50] and more than 90% of BC’s population speaks English [51], we are confident that this study will include a representative sample of THR patients. In our future full RCT, we aim to recruit patients from both urban and rural areas in BC to improve the generalizability of HIPPER. Despite developing the HIPPER modules with a user-centered approach, it is plausible that eHealth approaches may favor younger cohorts with greater computer experience and discourage older adults from participating [52]. While this assumption will be explored statistically, our patient partners’ suggestions for “marketing” to older adults and adding basic computer training in the module introduction may address this issue. We will try to minimize barriers related to technology by sending each participant a guideline on how to use HIPPER, encouraging participants to contact the research assistant for further instruction, and inviting family caregivers to help participants use the online modules. In addition, in the current study, it is not possible to blind the participants to their group as it will be clear to participants what type of intervention they are receiving. Therefore, as other studies have suggested, the effect size might be substantially higher in trials in which participants are not blinded [53,54]. As recommended by previous research [55], to overcome the lack of participants’ blinding, participants who previously attended OASIS webinars or in-person education will be excluded. In addition, participants in the control group will be prevented from accessing the HIPPER modules. Furthermore, the assessors in this study are blinded, and we will ask them to report any unblinding immediately. The assessors will be replaced if they report any potential or actual unblinding.

Finally, participants in the HIPPER group may be directed to the OASIS webinar educations by their surgeon, other health care providers, and other patients. Therefore, our educators at OASIS will be given the names of the participants and will be asked whether the participants attended any webinars. Participants in both HIPPER and control groups will also be asked at T2 whether they attended the OASIS webinars. Similar to previous studies [39], we anticipate a 20% dropout rate, meaning that participants will decline to participate in the study. If we encounter any dropout, we will recruit additional participants to keep the sample size for each group at 20.

### Future Research

This project is a feasibility and preparatory study. The findings will be used to develop a definitive RCT, which will help us test the impact of HIPPER in a larger sample size before making it widely available for patients undergoing THR. If HIPPER is found to be effective in the future trial, we will collaborate with OASIS to host HIPPER on their website and encourage broad dissemination and implementation throughout BC.

## Appendix

Multimedia Appendix 1 Feasibility indicators.

## Abbreviations

ANCOVA: analysis of covariance
BC: British Columbia
CST: Chair Stand Test
EQ-5D-5L: EuroQoL-5 Dimension, 5 Level
HADS-A: Hospital Anxiety and Depression Scale
HIPPER: Hip Instructional Prehabilitation Program for Enhanced Recovery
OA: osteoarthritis
OASIS: OsteoArthritis Service Integration System
OHS: Oxford Hip Score
PASE: Physical Activity Scale for the Elderly
prehab: Prehabilitation
RCT: randomized controlled trial
SER: Self-Efficacy for Rehabilitation Outcome Scale
SPIRIT: Standard Protocol Items: Recommendations for International Trials)
THR: total hip replacement

## References

[1] Metcalfe S, Ju H, Molodianovitsh K, Sidhom P, Faye L, Webste G (2015). Knee Replacement in Canada: Canadian Joint Replacement Registry 2015 Annual Report. Canadian Institute for Health Information.

[2] Ruchlin H, Elkin E, Allegrante J (2001). The economic impact of a multifactorial intervention to improve postoperative rehabilitation of hip fracture patients. Arthritis & Rheumatism.

[3] Santavirta N, Lillqvist G, Sarvimäki A, Honkanen V, Konttinen Y, Santavirta S (1994). Teaching of patients undergoing total hip replacement surgery. Int J Nurs Stud.

[4] Butler G, Hurley C, Buchanan K, Smith-VanHorne J (1996). Prehospital education: effectiveness with total hip replacement surgery patients. Patient Education and Counseling.

[5] Santa Mina D, Scheede-Bergdahl C, Gillis C, Carli F (2015). Optimization of surgical outcomes with prehabilitation. Appl Physiol Nutr Metab.

[6] Moulton LS, Evans PA, Starks I, Smith T (2015). Pre-operative education prior to elective hip arthroplasty surgery improves postoperative outcome. Int Orthop.

[7] Ibrahim M, Twaij H, Giebaly D, Nizam I, Haddad F (2013). Enhanced recovery in total hip replacement. The Bone & Joint Journal.

[8] Aydin D, Klit J, Jacobsen S, Troelsen A, Husted H (2015). No major effects of preoperative education in patients undergoing hip or knee replacement--a systematic review. Dan Med J.

[9] Westby M, Marshall D, Jones C (2018). Development of quality indicators for hip and knee arthroplasty rehabilitation. Osteoarthritis Cartilage.

[10] McDonald S, Page MJ, Beringer K, Wasiak J, Sprowson A (2014). Preoperative education for hip or knee replacement. Cochrane Database Syst Rev.

[11] Jayakumar N, Brunckhorst O, Dasgupta P, Khan MS, Ahmed K (2015). e-Learning in Surgical Education: A Systematic Review. J Surg Educ.

[12] Gruner D, Pottie K, Archibald D, Allison J, Sabourin V, Belcaid I, McCarthy A, Brindamour M, Augustincic Polec L, Duke P (2015). Introducing global health into the undergraduate medical school curriculum using an e-learning program: a mixed method pilot study. BMC Med Educ.

[13] van de Steeg L, IJkema R, Wagner C, Langelaan M (2015). The effect of an e-learning course on nursing staff's knowledge of delirium: a before-and-after study. BMC Med Educ.

[14] Parker SJ, Jessel S, Richardson JE, Reid MC (2013). Older adults are mobile too!Identifying the barriers and facilitators to older adults' use of mHealth for pain management. BMC Geriatr.

[15] Martin T (2012). Assessing mHealth: opportunities and barriers to patient engagement. J Health Care Poor Underserved.

[16] (2019). Internet use by location of use, age group, household income and geography. Statistics Canada.

[17] Knowles M (1978). The Adult Learner: A Neglected Species.

[18] Kujala S (2003). User involvement: A review of the benefits and challenges. Behaviour & Information Technology.

[19] Chan A, Tetzlaff J, Altman D, Laupacis A, Gøtzsche PC, Krleža-Jerić K, Hróbjartsson A, Mann H, Dickersin K, Berlin J, Doré CJ, Parulekar W, Summerskill W, Groves T, Schulz K, Sox H, Rockhold F, Rennie D, Moher D (2013). SPIRIT 2013 statement: defining standard protocol items for clinical trials. Ann Intern Med.

[20] Duivenvoorden T, Vissers M, Verhaar J, Busschbach J, Gosens T, Bloem R, Bierma-Zeinstra S, Reijman M (2013). Anxiety and depressive symptoms before and after total hip and knee arthroplasty: a prospective multicentre study. Osteoarthritis Cartilage.

[21] Rolfson O, Wissig S, van Maasakkers L, Stowell C, Ackerman I, Ayers D, Barber T, Benzakour T, Bozic K, Budhiparama N, Caillouette J, Conaghan PG, Dahlberg L, Dunn J, Grady-Benson J, Ibrahim SA, Lewis S, Malchau H, Manzary M, March L, Nassif N, Nelissen R, Smith N, Franklin PD (2016). Defining an International Standard Set of Outcome Measures for Patients With Hip or Knee Osteoarthritis: Consensus of the International Consortium for Health Outcomes Measurement Hip and Knee Osteoarthritis Working Group. Arthritis Care Res (Hoboken).

[22] Dobson F, Hinman R, Roos E, Abbott J, Stratford P, Davis A, Buchbinder R, Snyder-Mackler L, Henrotin Y, Thumboo J, Hansen P, Bennell K (2013). OARSI recommended performance-based tests to assess physical function in people diagnosed with hip or knee osteoarthritis. Osteoarthritis Cartilage.

[23] Nilsdotter A, Bremander A (2011). Measures of hip function and symptoms: Harris Hip Score (HHS), Hip Disability and Osteoarthritis Outcome Score (HOOS), Oxford Hip Score (OHS), Lequesne Index of Severity for Osteoarthritis of the Hip (LISOH), and American Academy of Orthopedic Surgeons (AAOS) Hip and Knee Questionnaire. Arthritis Care Res (Hoboken).

[24] Naal FD, Sieverding M, Impellizzeri FM, von Knoch F, Mannion AF, Leunig M (2009). Reliability and validity of the cross-culturally adapted German Oxford hip score. Clin Orthop Relat Res.

[25] Gill S, McBurney H (2008). Reliability of performance-based measures in people awaiting joint replacement surgery of the hip or knee. Physiother Res Int.

[26] Gill SD, de Morton NA, Mc Burney H (2012). An investigation of the validity of six measures of physical function in people awaiting joint replacement surgery of the hip or knee. Clin Rehabil.

[27] Washburn RA, McAuley E, Katula J, Mihalko SL, Boileau RA (1999). The physical activity scale for the elderly (PASE): evidence for validity. J Clin Epidemiol.

[28] Svege I, Kolle E, Risberg MA (2012). Reliability and validity of the Physical Activity Scale for the Elderly (PASE) in patients with hip osteoarthritis. BMC Musculoskelet Disord.

[29] Waldrop D, Lightsey ORJ, Ethington CA, Woemmel CA, Coke AL (2001). Self-efficacy, optimism, health competence, and recovery from orthopedic surgery. Journal of Counseling Psychology.

[30] Stevens M, van den Akker-Scheek I, van Horn JR (2005). A Dutch translation of the Self-Efficacy for Rehabilitation Outcome Scale (SER): a first impression on reliability and validity. Patient Educ Couns.

[31] EuroQol Group (1990). EuroQol--a new facility for the measurement of health-related quality of life. Health Policy.

[32] Brooke J, Jordan PW, Thomas B, McClelland BA, Weerdmeester LI (1996). SUS: A quickdirty usability scale. Usability Evaluation in Industry.

[33] Billingham S, Whitehead A, Julious S (2013). An audit of sample sizes for pilot and feasibility trials being undertaken in the United Kingdom registered in the United Kingdom Clinical Research Network database. BMC Med Res Methodol.

[34] Julious SA (2005). Sample size of 12 per group rule of thumb for a pilot study. Pharmaceut. Statist.

[35] Vranceanu A, Jacobs C, Lin A, Greenberg J, Funes CJ, Harris MB, Heng MM, Macklin EA, Ring D (2019). Results of a feasibility randomized controlled trial (RCT) of the Toolkit for Optimal Recovery (TOR): a live video program to prevent chronic pain in at-risk adults with orthopedic injuries. Pilot Feasibility Stud.

[36] Singal A, Higgins P, Waljee A (2014). A primer on effectiveness and efficacy trials. Clin Transl Gastroenterol.

[37] Arain M, Campbell MJ, Cooper CL, Lancaster GA (2010). What is a pilot or feasibility study? A review of current practice and editorial policy. BMC Med Res Methodol.

[38] Randomisation and Online Databases for Clinical Trials. Sealed Envelope.

[39] Brosseau L, Wells G, Brooks-Lineker S, Bennell K, Sherrington C, Briggs A, Sturnieks D, King J, Thomas R, Egan M, Loew L, De Angelis G, Casimiro L, Toupin April K, Cavallo S, Bell M, Ahmed R, Coyle D, Poitras S, Smith C, Pugh A, Rahman P (2015). Internet-based implementation of non-pharmacological interventions of the "people getting a grip on arthritis" educational program: an international online knowledge translation randomized controlled trial design protocol. JMIR Res Protoc.

[40] Borm GF, Fransen J, Lemmens WA (2007). A simple sample size formula for analysis of covariance in randomized clinical trials. J Clin Epidemiol.

[41] Dimitrov D, Rumrill P (2003). Pretest-posttest designs and measurement of change. Work.

[42] Overall JE, Spiegel DK (1969). Concerning least squares analysis of experimental data. Psychological Bulletin.

[43] Moncur RA, Larmer JC (2009). Clinical applicability of intention-to-treat analyses. Evidence Based Medicine.

[44] DeMets D (1998). Datasafety monitoring boards. Armitage P, Colton T. editors.

[45] OsteoArthritis Service Integration System (OASIS). Vancouver Coastal Health.

[46] Jayakumar N, Brunckhorst O, Dasgupta P, Khan MS, Ahmed K (2015). e-Learning in Surgical Education: A Systematic Review. J Surg Educ.

[47] Dear BF, Zou JB, Ali S, Lorian CN, Johnston L, Terides MD, Staples LG, Gandy M, Fogliati VJ, Klein B, Titov N (2015). Examining self-guided internet-delivered cognitive behavior therapy for older adults with symptoms of anxiety and depression: Two feasibility open trials. Internet Interventions.

[48] Alley SJ, Kolt GS, Duncan MJ, Caperchione CM, Savage TN, Maeder AJ, Rosenkranz RR, Tague R, Van Itallie AK, Kerry Mummery W, Vandelanotte C (2018). The effectiveness of a web 2.0 physical activity intervention in older adults - a randomised controlled trial. Int J Behav Nutr Phys Act.

[49] (2019). Hip and Knee Replacements in Canada, 2017-2018: Canadian Joint Replacement Registry Annual Report Internet. Canadian Institute for Health Research.

[50] Canada IFHI Hip and Knee Replacements in Canada: Canadian Joint Replacement Registry 2015 Annual Report Internet. Canada Institute for Health Information.

[51] (2012). Visual Census – Language, Vancouver. Statistics Canada.

[52] Tiedeman DV, Knowles M (1979). The Adult Learner: A Neglected Species. Educational Researcher.

[53] Hróbjartsson A, Emanuelsson F, Skou Thomsen AS, Hilden J, Brorson S (2014). Bias due to lack of patient blinding in clinical trials. A systematic review of trials randomizing patients to blind and nonblind sub-studies. Int J Epidemiol.

[54] Day S, Altman D (2000). Statistics notes: blinding in clinical trials and other studies. BMJ.

[55] Deyo RA, Walsh NE, Schoenfeld LS, Ramamurthy S (1990). Can trials of physical treatments be blinded? The example of transcutaneous electrical nerve stimulation for chronic pain. Am J Phys Med Rehabil.

