# Correction: The Hip Instructional Prehabilitation Program for Enhanced Recovery (HIPPER) as an eHealth Approach to Presurgical Hip Replacement Education: Protocol for a Randomized Controlled Trial

**DOI:** 10.2196/39745

**Published:** 2022-07-14

**Authors:** William C Miller, Somayyeh Mohammadi, Wendy Watson, Morag Crocker, Marie Westby

**Affiliations:** 1 GF Strong Rehabilitation Research Program Vancouver, BC Canada; 2 Department of Occupational Science and Occupational Therapy Faculty of Medicine University of British Columbia Vancouver, BC Canada; 3 Vancouver Coastal Health Vancouver, BC Canada

In “The Hip Instructional Prehabilitation Program for Enhanced Recovery (HIPPER) as an eHealth Approach to Presurgical Hip Replacement Education: Protocol for a Randomized Controlled Trial” (JMIR Res Protoc 2021;10(7):e29322) the authors noted the following errors:

The fourth timepoint (T4) was not recorded as a part of the study and was inadvertently included in the paper. As a result, the following 17 corrections have been made:

1. In the originally published article, the following indicator appeared in “Table 1. Feasibility Indicators” as a retention rate process indicator:

% of participants with T4^a^ data

This has been corrected to:

% of participants with T3^a^ data

2. In the originally published article, the following indicator appeared in “Table 1. Feasibility Indicators” as a perceived benefit process indicator:

Posttreatment participant questionnaire; qualitative interviews at T4

This has been corrected to:

Posttreatment participant questionnaire; qualitative interviews at T3

3. In the originally published article, the following indicator appeared in “Table 1. Feasibility Indicators” as a data collection (T): participant & assessor burden resources indicator:

T1 duration; T2, T3, & T4 durations

This has been corrected to:

T1 duration; T2, & T3 durations

4. In the originally published article, the following footnote appeared in “Table 1. Feasibility Indicators”:

^a^Fourth measurement timepoint.

This has been corrected to:

^a^Third measurement timepoint.

5. In the originally published article, the following sentence was included in the “Secondary Outcomes” section regarding the Oxford Hip Score:

Participants will complete this measure at all time points (T1-T4).

This sentence has been corrected to:

Participants will complete this measure at all time points (T1-T3).

6. In the originally published article, the following sentence was included in the “Secondary Outcomes” section regarding the 30-second Chair Stand Test:

Participants will be asked to complete this test at T1, T2, and T4.

This sentence has been corrected to:

Participants will be asked to complete this test at T1 and T2.

7. In the originally published article, the following sentence was included in the “Secondary Outcomes” section regarding the Physical Activity Scale for the Elderly:

Participants will complete this measure at T1-T4.

This sentence has been corrected to:

Participants will complete this measure at T1-T3.

8. In the originally published article, the following sentence was included in the “Secondary Outcomes” section regarding the Self-Efficacy for Rehabilitation Outcome Scale:

Participants will complete this measure at T1-T4.

This sentence has been corrected to:

Participants will complete this measure at T1-T3.

9. In the originally published article, the following sentences were included in the “Secondary Outcomes” section regarding the equipment checklist:

Patients will use the checklist at T2-T4 to record the number and type of equipment items they have used and how often they have used each (eg, dressing equipment). Participants will complete this measure at T2-T4.

These sentences have been corrected to:

Patients will use the checklist at T2 and T3 to record the number and type of equipment items they have used and how often they have used each (eg, dressing equipment). Participants will complete this measure at T2-T3.

10. In the originally published article, the following sentence was included in the “Secondary Outcomes” section regarding the EuroQoL-5 Dimension, 5 Level:

Participants will complete this measure at T1-T4.

This sentence has been corrected to:

Participants will complete this measure at T1-T3.

11. In the originally published article, the following sentence was included in the “Secondary Outcomes” section regarding the System Usability Scale:

Participants will complete this measure at T2-T4 (if randomized to the treatment group).

This sentence has been corrected to:

Participants will complete this measure at T2 and T3 (if randomized to the treatment group).

12. In the originally published article, the following sentence was included in the “Participant Timeline” section:

Follow-up data will be collected 7-10 days prior to surgery (T2), 30 days (T3) postsurgery, and 90 days (T4) postsurgery.

This sentence has been corrected to:

Follow-up data will be collected 7-10 days prior to surgery (T2), 30 days and (T3) postsurgery.

13. In the originally published article, the following sentences were included in the ”Data Collection Method and Data Management“ section:

To collect the data at T2, T3, and T4, the assessor will send a link to the participants to complete the questionnaires in Qualtrics. In addition, at T2 and T4, the assessor will contact the participants to schedule a short online meeting during which the participants will be asked to do the 30-sec CST via videoconference.

These sentences have been corrected to:

To collect the data at T2 and T3, the assessor will send a link to the participants to complete the questionnaires in Qualtrics. In addition, at T2 the assessor will contact the participants to schedule a short online meeting during which the participants will be asked to do the 30-sec CST via videoconference.

14. In the originally published article, Multimedia Appendix 1 indicated ”Retention rate“ process indicator indicated:

% of participants with T4 data

This has been corrected to:

% of participants with T3 data

15. In the originally published article, Multimedia Appendix 1 indicated “Perceived benefit” process indicator indicated:

Qualitative Interviews at T4*

This has been corrected to:

Qualitative Interviews at T3*

16. In the originally published article, Multimedia Appendix 1 indicated “Data collection (T): Participant & Assessor burden” resources indicator indicated:

T2, 3 & 4 duration

This has been corrected to:

*T2 & 3 duration*

The revised version of the appendix is in Multimedia Appendix 1.

17. In the originally published article, Figure 1 contained a box indicating “T4 Data Collection 90 days post surgery” (see Multimedia Appendix 2).

In the corrected article, Figure 1 has been corrected by removing this box as follows:

**Figure 1:**
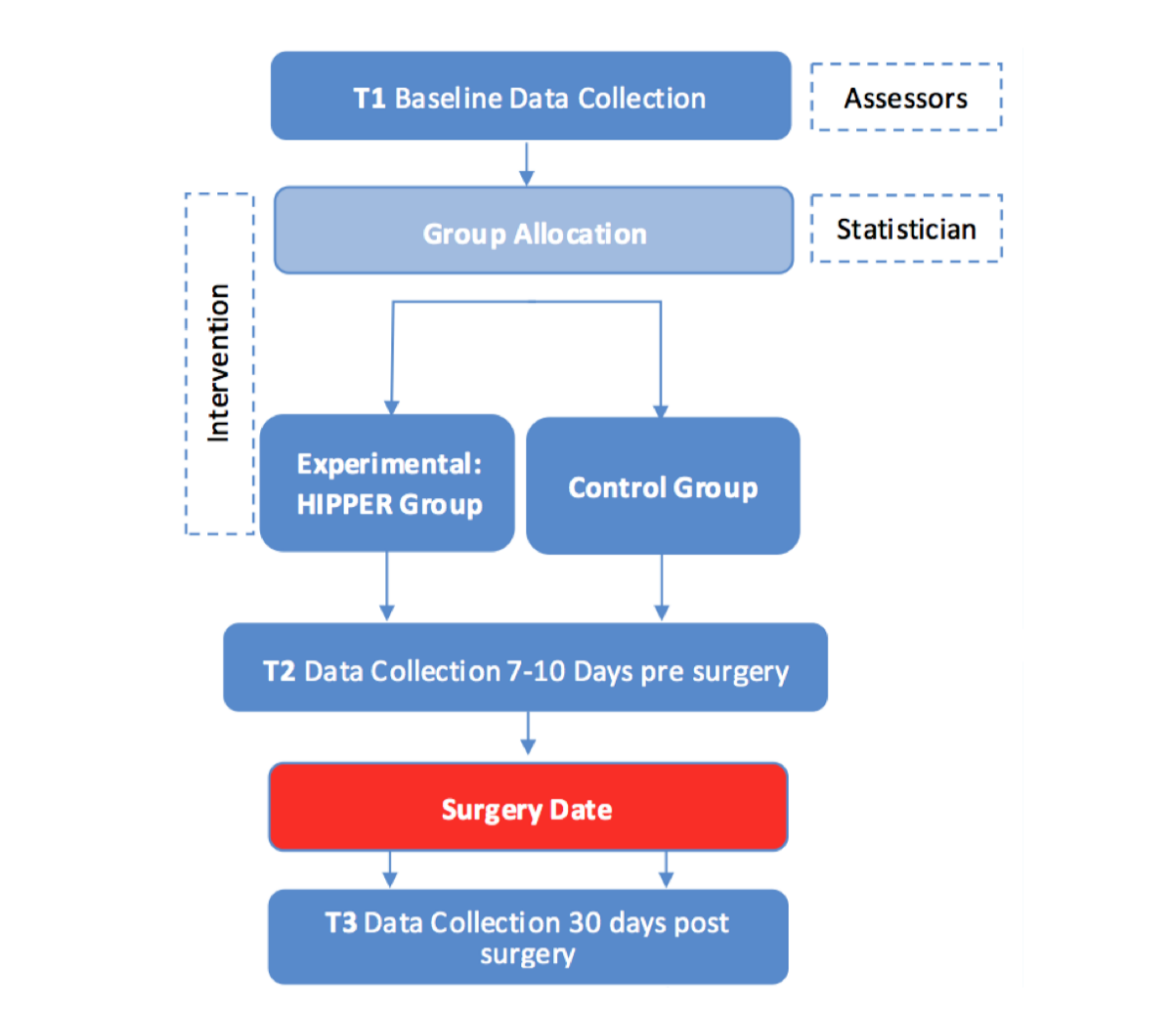
Data collection procedure for the randomized controlled trial. HIPPER: Hip Instructional Prehabilitation Program for Enhanced Recovery.

The correction will appear in the online version of the paper on the JMIR Publications website on July 14, 2022, together with the publication of this correction notice. Because this was made after submission to PubMed, PubMed Central, and other full-text repositories, the corrected article has also been resubmitted to those repositories.

## Appendix

Multimedia Appendix 1 Feasibility indicators.

Multimedia Appendix 2 Originally published version of Figure 1.

