# Supplementary material for: The Hip Instructional Prehabilitation Program for Enhanced Recovery (HIPPER) as an eHealth Approach to Presurgical Hip Replacement Education: Protocol for a Randomized Controlled Trial
Source: JMIR Res Protoc. 2021 Jul 6;10(7):e29322. doi: 10.2196/29322 (PMC8292937; doi:10.2196/29322)
Supplement: Multimedia Appendix 1 [file resprot_v10i7e29322_app1.docx]

|  | Feasibility Component | Indicator | **Criteria** |
| --- | --- | --- | --- |
| ***Process*** | Recruitment rate | # of participants recruited  # of women and men recruited | Mean of 4 participants/month: Total of 44 over 11 months |
|  | Consent rate | % of participants consenting | < 10% participant refusal |
|  | Retention rate | % of participants with T4 data | Complete data collection for > 80% |
|  | Perceived benefit | Post-treatment Participant Questionnaire  Qualitative Interviews at T4* | > 85% of responses will be “strongly agree/agree”  Qualitative analysis will inform clinical importance |
|  | Assessor masking | % unaware of group status | 100% of participants do not unmask their treatment |
| ***Resources*** | Treatment adherence  (HIPPER)  (Control Group) | Attend to all modules  2.5 hours spent all modules  Attend both prehab education sessions | > 85% of participants  > 85% of participants  > 85% of participants |
|  | Data collection (T):  Participant & Assessor burden | T1 duration  T2, 3 & 4 duration | > 85% of participants complete in ≤2h  > 85% of participants complete in ≤1.5h |
|  | Collection of EQ-5D data | Administration  EQ-5D pre/post score | Mean EQ-5D administration is <10 min  Statistically significant change between T1 & T2 |
|  | Educator burden | Time (minutes) spent in answering participants’ questions and following up with them | Mean time spent per participant is <2 hours for T1 and <1 hour for T2  < 20% phone call back for clarification |
| ***Management*** | Internet Stability | Downtime due to technical or mechanical issues | > 90% of participants are not without internet for > 2 days |
|  | Participant processing time | Time from data collection to treatment | Mean time is < 10 days at each site |
|  | Treatment administration issues | Post-treatment Evaluation Form (Study educator) | Any issues identified modifiable without substantial changes to the protocol |
| ***Treatment*** | Safety (Data Collection & Training) | Adverse events during assessment or training | No major injuries or adverse events (e.g. dislocation) reported |
|  | Dose level response | Correlation between training time and change score | Minimum practice time guidelines sufficient for a treatment effect |
