# Supplementary figures and images for: Correction: The Hip Instructional Prehabilitation Program for Enhanced Recovery (HIPPER) as an eHealth Approach to Presurgical Hip Replacement Education: Protocol for a Randomized Controlled Trial
Source: JMIR Res Protoc. 2022 Jul 14;11(7):e39745. doi: 10.2196/39745 (PMC9335173; doi:10.2196/39745)

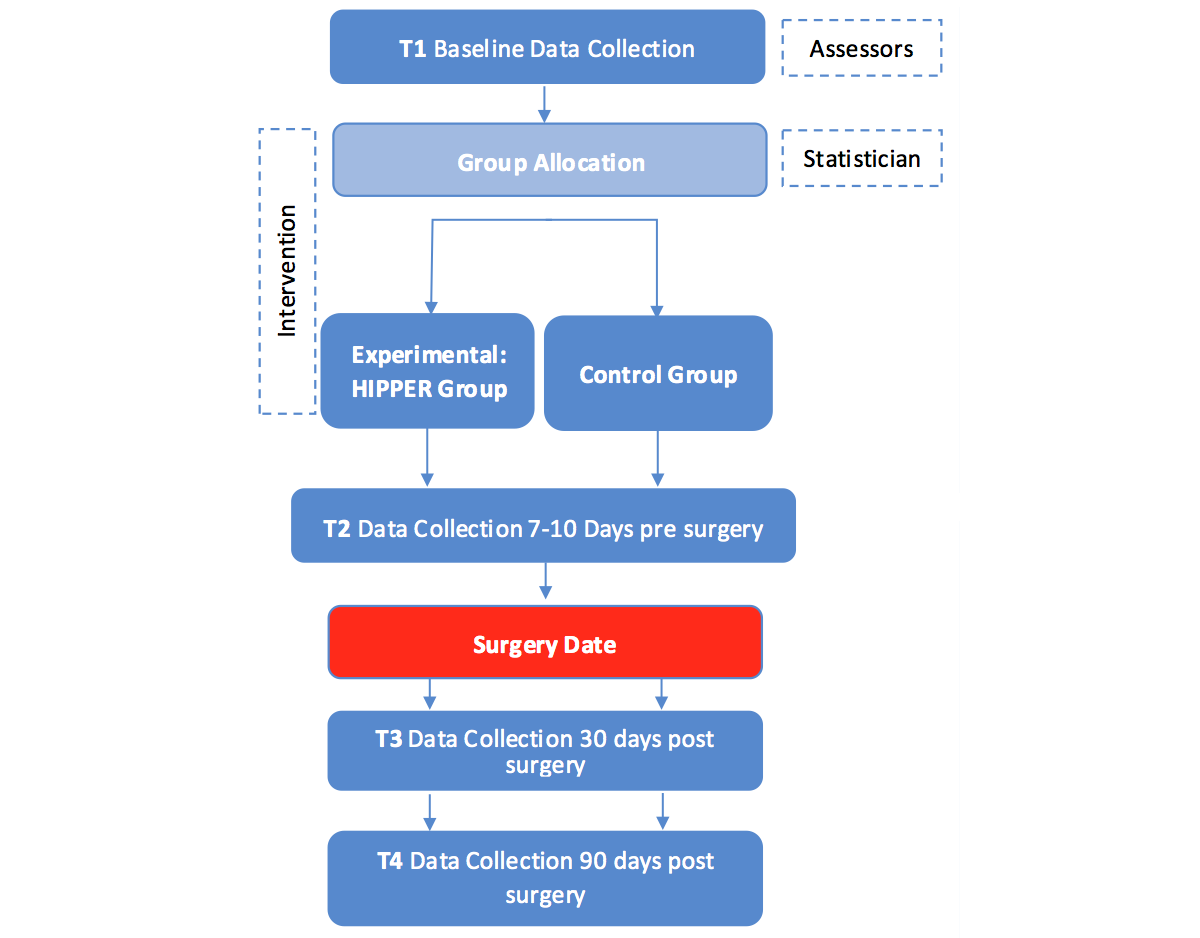

Supplement: Multimedia Appendix 2 [file resprot_v11i7e39745_app2.png]
